# Experimental validation of predicted subcellular localizations of human proteins

**DOI:** 10.1186/1756-0500-7-912

**Published:** 2014-12-15

**Authors:** Nagendra K Chaturvedi, Riyaz A Mir, Vimla Band, Shantaram S Joshi, Chittibabu Guda

**Affiliations:** Department of Genetics, Cell Biology and Anatomy, University of Nebraska Medical Center, 985870 Nebraska Medical Center, Omaha, NE 68198 USA; Fred and Pamela Buffet Cancer Center, Omaha, USA; Eppley Institute for Cancer Research, Omaha, USA; Bioinformatics and Systems Biology Core, University of Nebraska Medical Center, Omaha, NE 68198-5805 USA

**Keywords:** Protein subcellular localization, ngLOC prediction, Gene cloning, Experimental validation, GFP fusion, Live cell imaging/confocal microscopy

## Abstract

**Background:**

Computational methods have been widely used for the prediction of protein subcellular localization. However, these predictions are rarely validated experimentally and as a result remain questionable. Therefore, experimental validation of the predicted localizations is needed to assess the accuracy of predictions so that such methods can be confidently used to annotate the proteins of unknown localization. Previously, we published a method called ngLOC that predicts the localization of proteins targeted to ten different subcellular organelles. In this short report, we describe the accuracy of these predictions using experimental validations.

**Findings:**

We have experimentally validated the predicted subcellular localizations of 114 human proteins corresponding to nine different organelles in normal breast and breast cancer cell lines using live cell imaging/confocal microscopy. Target genes were cloned into expression vectors as GFP fusions and cotransfected with RFP-tagged organelle-specific gene marker into normal breast epithelial and breast cancer cell lines. Subcellular localization of each target protein is confirmed by colocalization with a co-expressed organelle-specific protein marker. Our results showed that about 82.5% of the predicted subcellular localizations coincided with the experimentally validated localizations. The highest agreement was found in the endoplasmic reticulum proteins, while the cytoplasmic location showed the least concordance. With the exclusion of cytoplasmic location, the average prediction accuracy increased to 90.4%. In addition, there was no difference observed in the protein subcellular localization between normal and cancer breast cell lines.

**Conclusions:**

The experimentally validated accuracy of ngLOC method with (82.5%) or without cytoplasmic location (90.4%) nears the prediction accuracy of 89%. These results demonstrate that the ngLOC method can be very useful for large-scale annotation of the unknown subcellular localization of proteins.

**Electronic supplementary material:**

The online version of this article (doi:10.1186/1756-0500-7-912) contains supplementary material, which is available to authorized users.

## Findings

### Background

Subcellular localization of proteins to specific compartments is fundamental to the structural organization and functioning of all living cells. Proteins that are localized to unintended organelles have been implicated in the development of many human diseases; therefore, knowledge of the protein subcellular localization can benefit target identification in the drug discovery process [1].

Protein subcellular localization is an important attribute of protein function; thus, prediction of the same aids in genome annotation of high-throughput studies. Numerous computational methods have been used for the prediction of proteins subcellular localization [2]. Among these, some are limited by predicting only a small number of organelles in the cell [3, 4] while some others exhibit lack of a balance between sensitivity and specificity [5, 6]. Previously, we have developed a method called ngLOC, an *n*-gram based Bayesian method that can predict a wide range of subcellular locations including multiple localizations of proteins [7, 8]. This method makes its predictions solely based on the protein sequence information without the need for any extraneous information; therefore ngLOC is highly favorable for proteome-wide prediction of subcellular localizations.

The ngLOC method predicts subcellular locations at a high overall accuracy of 89%, while the accuracy is much higher (93-96%) in organelles with smaller proteomes such as lysosomes, peroxisomes, Golgi, etc., [7] that are typically difficult to predict due to lack of sufficient size datasets. Although computational predictions provide wealth of information for the subcellular localization of proteins, these predictions remain questionable unless they are validated by experimental methods. In the present study, we report experimental validations for ngLOC predicted subcellular localizations of human proteins. Our results corroborated the predicted results; thus ngLOC method can be used for proteome-wide annotation of protein localizations.

### Materials and methods

#### Reagents and materials

Restriction enzymes and DH5α-competent cells were purchased from New England Biolabs (MA, USA). Trizol™, transfection reagent Lipofectamine2000^TM^, and red fluorescent tagged-subcellular markers including Mitotracker™ Red FM, Lysotracker™ Red, ER-Tracker™ Red, BODIPY® TR ceramide, Hoechst 33342 and Alexa Fluor® 594 WGA were obtained from Invitrogen (CA, USA). A cDNA synthesis and ligation kit was purchased from Promega (WI, USA). Primers of all cloned genes for the PCR amplification were obtained from Integrated DNA Technologies Inc. (Coralville, IA). A 2X PCR amplification kit was purchased from Applied Biological Materials Inc. (Richmond, Canada). Plasmid and DNA gel extraction kits were obtained from Qiagen Inc. (Valencia, CA). Fluorodish 35 mm petriplates for live cell imaging were purchased from World Precision Instruments (Sarasota, FL). All plastic wares for mammalian cell culture were purchased from Corning Costar Corp. (NY, USA).

#### Plasmids and constructs

pEGFP-N1 vector was kindly provided by Dr. Hamid Band (UNMC). Ten GFP-tagged full-length human gene constructs (ngLOC predicted), which include: SACM1L, ST13, TUBAL3, USMG5, DECR2, AMY2B, UXS1, LGMN, NR2F1 and NAPB were obtained from Origene Technologies Inc. (Rockville, MD). Six RFP-tagged subcellular specific human gene constructs (positive markers) which include: endoplasmic reticulum specific ETS, Golgi specific TGOLN, peroxisome specific PXPM2, mitochondria specific PDHA1, plasma membrane specific LCK and cytoskeleton specific β-ACTIN were also purchased from Origene Technologies Inc. (Rockville, MD).

#### Isolation of RNA and cDNA preparation

Total RNA was extracted from HEK-293 T cells using the TRIzol™ method according to the manufacturer’s instructions. RNA quantity and purity were determined by UV spectrophotometry and by electrophoresis on a 2% agarose gel. Two micrograms of RNA was then reverse transcribed using random hexamer primers and the superscript RT enzyme according to the manufacturer’s instructions (Invitrogen, CA).

#### PCR amplification and gene cloning

PCR Amplification was achieved with the 2X PCR master mix kit containing Taq DNA polymerase using 30–35 cycles according to the manufacturer’s protocols. For amplification, the two sets of primers with appropriate restriction enzymes were used against full-length ORF of each human gene. The primers used for the genes cloning of this study have been tabulated in Additional file 1. Each PCR amplified gene product was separated on 1% agarose gel in 1X TAE buffer (pH 8.0) and visualized by ethidium bromide staining. The gel extraction of PCR amplified gene products were purified using a gel extraction kit; then these purified genes products were double digested with restriction enzymes using the combination of either NheI/XhoI, NheI/HindIII or BglII/BamHI. Following restriction digestions, the full-length genes were cloned into a pEGFP-N1 vector using a LigaFast ligation kit and were transformed into E. *coli* (DH5α) bacterial strain. The positive clones of the genes were screened and confirmed, following appropriate restriction digestion.

#### Cell lines and culture conditions

The normal breast epithelial cell lines MCF-10A and MCF-12 F were obtained from the American Type of Culture Collection (Rockville, MD). These cell lines were maintained in D-media described previously [9]. The breast cancer epithelial cell lines MCF-7 and MDA-MB-231 were kindly provided by Dr. Vimla band (UNMC). These cells were cultured in α-MEM media supplemented with 10% FBS (Invitrogen, CA), 2 mM glutamine (Invitrogen, CA), 50 μg/ml gentamicin (Invitrogen CA), 1x sodium pyruvate (Invitrogen CA), 1x MEM non-essential amino acid (Invitrogen, CA), 1x HEPES (Invitrogen, CA) and 1 μg/ml insulin (Sigma). The cultures were maintained in a humidified incubator adjusted at 5% CO2 and 95% air atmosphere at 37°C. All cultures were passaged twice a week and maintained at a concentration no greater than 1 × 10^6^/ml.

#### Transient transfections and confocal microscopy

Breast normal (MCF-10A, MCF-12 F) and cancer (MCF-7, MDA-MB-231) epithelial cells were seeded on 35-mm fluorodish petriplates to reach approximately 50-70% confluence in their respective medium. The next day, cells were transiently co-transfected with 1 μg of GFP-tagged predicted target gene and subcellular specific RFP-tagged marker gene (endoplasmic reticulum specific ETS, Golgi specific TGOLN, peroxisome specific PXPM2, mitochondria specific PDHA1, plasma membrane specific LCK and cytoskeleton specific β-ACTIN) for each of the localizations, using Lipofectamine in serum free MEM medium. After 6–8 hours of transfection incubation, cells were supplemented with a complete respective media and given another 12 hours of incubation for the protein expression. Following protein expression, subcellular distribution and co-localization of proteins were assessed under the confocal microscope. Alternatively, other red fluorescent subcellular specific markers (dye) were also used with live cells to validate the each localization. Each predicted localization was confirmed and validated when the co-localization produces a yellow color upon merging the images of specific subcellular markers. Nuclear stain Hoechst-33342 (1 μg/ml) was added to live cells for the visualization of nucleus. Fluorescence images of live cells were recorded through Zeiss LSM 710 confocal microscope (Jena, Germany) with 40X objective lens. Images were captured and analyzed with LSM software (Jena, Germany) and processed using standard software programs.

### Results and discussion

**Figure 1:**
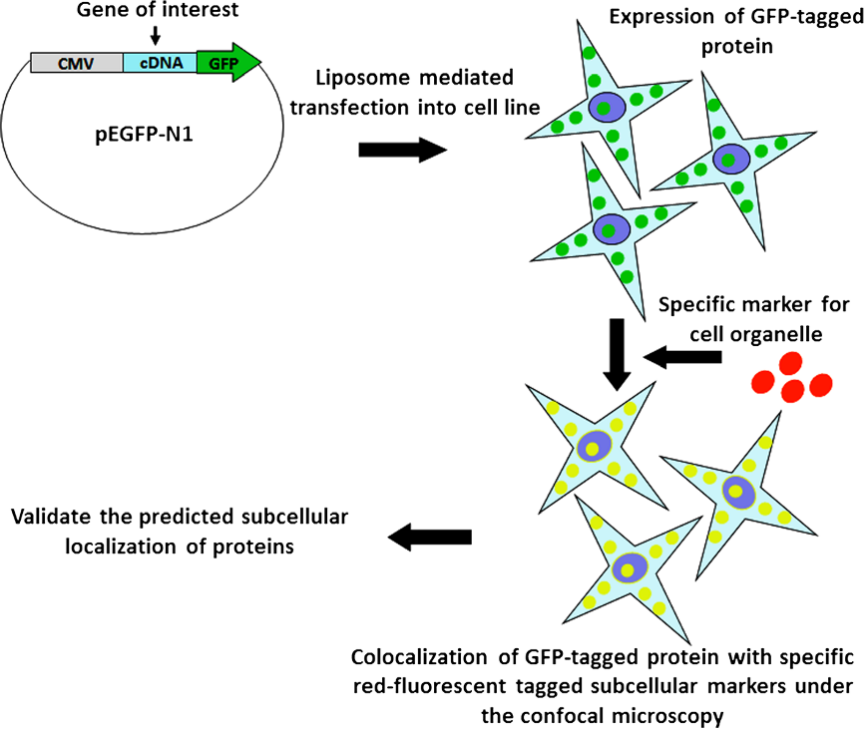
**The cartoon shows the research strategy used to experimentally validate the predicted subcellular localization of proteins.** Genes of interest were cloned into pEGP-N1 vector as GFP fusions. These GFP-tagged genes along with corresponding location-specific RFP-tagged gene markers, were transiently co-transfected using liposome-mediated method into two normal breast and breast cancer cell lines. Following 24 hours of transfection and protein expression, subcellular localization was determined using live cell imaging/confocal microscopy. Protein localizations to each compartment were confirmed by observing the colocalization of GFP- and RFP-tagged proteins that gives yellow color up on merging green and red images.

**Figure 2:**
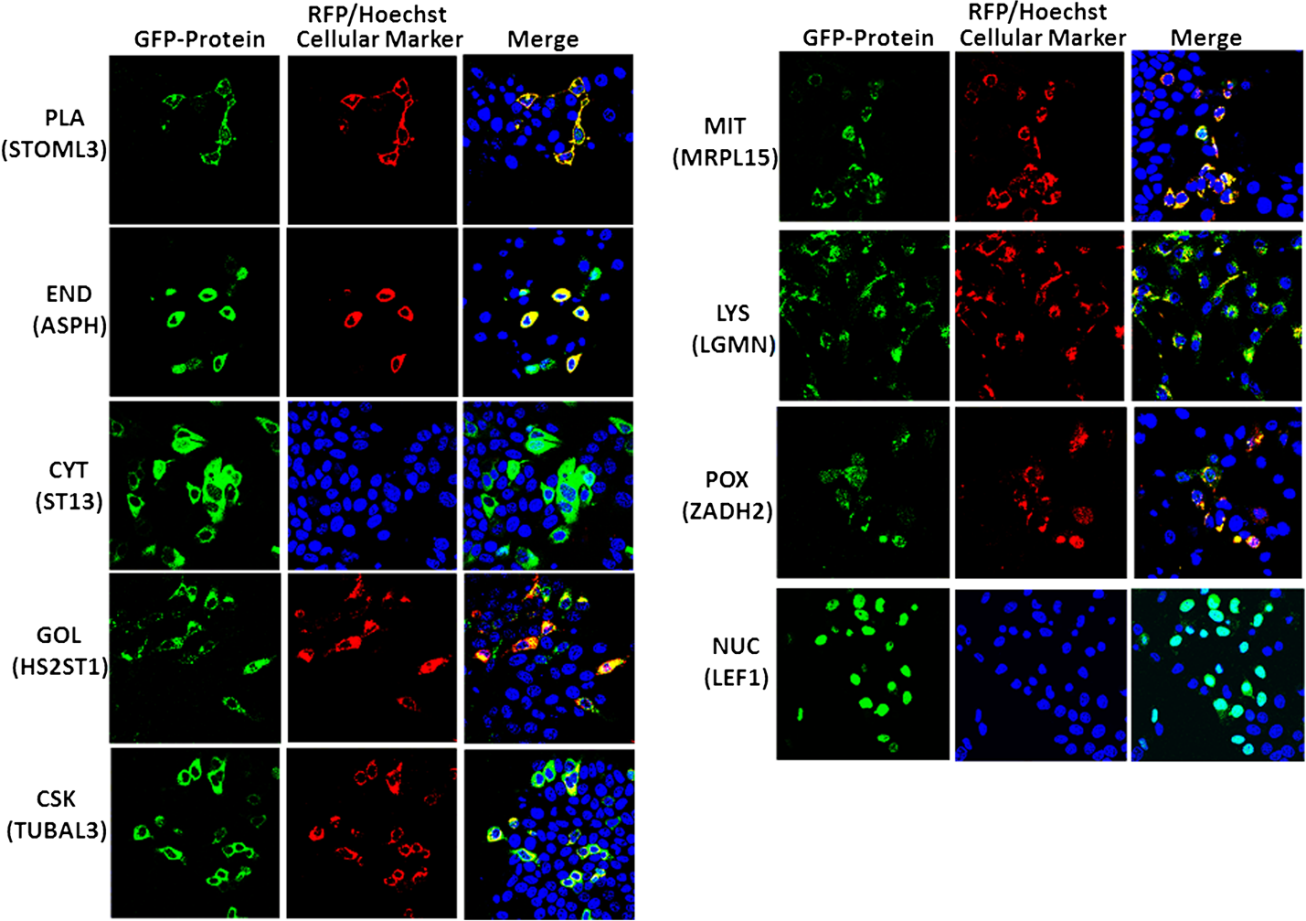
**Experimental validation of predicted localization of human proteins.** GFP-tagged full-length genes of target proteins and RFP-tagged compartment specific genes were transiently co-expressed in two normal and two breast cancer cell lines. Subcellular localization of the transiently expressed proteins was determined under the confocal microscope (40X). To facilitate the visualization of predicted subcellular localization, the specific RFP-tagged protein marker/dye for each localization was used in colocalization studies. Hoechst (nuclear dye) was used in all experiments. This figure shows a representative observation of colocalization in MCF-7 cells for each of the nine subcellular compartments used for validation in this study.

The research strategy used for experimental validation of ngLOC predicted protein subcellular localizations is described in Figure 1. cDNA was synthesized from HEK-293 T cells; with the use of cDNA, the genes of 105 target proteins of human origin were PCR amplified and then cloned into a GFP expression vector (pEGFP-N1) with GFP at the N-terminus as a fusion gene. Using the ngLOC method, 114 target proteins with predicted subcellular localization (includes 105 locally cloned and nine commercially obtained) were selected for this validation study (Additional file 2). GFP expressing fusion genes along with corresponding location-specific RFP-tagged protein markers, were transiently co-expressed following gene transfection into two normal breast (MCF-10A, MCF-12 F) and two breast cancer (MCF-7, MDA-231) cell lines; then their subcellular localization was determined using live cell imaging/confocal microscopy. In the present study, nine different subcellular compartments were selected for validating the predicted subcellular localization of proteins. The images in Figure 2 show a representation of validated localizations for predicted proteins in each compartment. The localizations for each compartment (except for nucleus and cytoplasm) were determined by observing the colocalization of GFP- and RFP-tagged proteins, which produced a yellow color upon merging the images. For the nucleus and cytoplasm, we used a nuclear (Hoechst) stain to validate the protein subcellular localization in either location (Figure 2).

**Figure 3:**
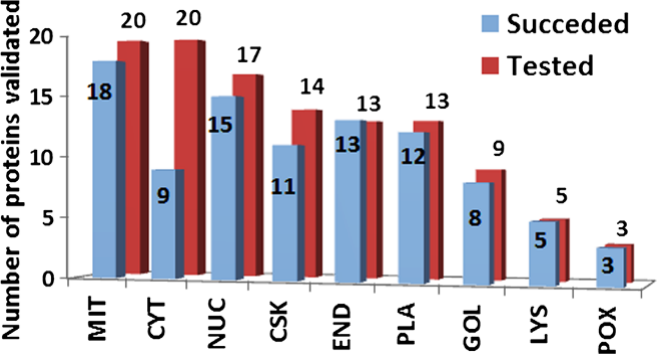
**Graph showing the total number of tested and those that are in agreement with the predicted localizations in each subcellular location.** This graph was generated based on the data provided in Table 1.

Table 1 lists the prediction for each gene tested, along with the outcome of the validation experiment. Similarly, Figure 3 shows the number of tested and succeeded proteins in the validation experiments. Our live cell imaging results showed that overall about 82.5% (94 out of 114) of proteins validated in this study agreed with the ngLOC predicted localizations; these results were consistent in all four cell lines tested. However, with the exclusion of the cytoplasm location that shows the lowest accuracy (45%), the average prediction rate increases to 90.4% (85 out of 94). ngLOC method outputs the predictions in a ranked order by using the associated confidence score (probability) for each location. The top two locations can be predicted within a close confidence range, suggesting that either or both of the predictions can be true. It is known that a number of proteins are localized to multiple organelles in eukaryotic cells (7). To test the accuracy of the second choice we also validated the second predictions of 30 proteins, which included 17 proteins (Set I) whose first choice predictions were proven wrong and 13 proteins (Set II) whose first choice predictions were accurate in the above experiments. From Set I, 10 proteins have shown homogenous distribution in cells, suggesting their localization both in the cytoplasm and nucleus (Table 2). For seven of these 10 proteins, the top two ngLOC predictions were cytoplasm or nucleus, which support our results that these proteins are localized in both nucleus and cytoplasm. From the other 7 proteins in Set I, the second choice predictions were validated as correct only for 2 proteins (Table 2). Validation results on Set II showed that about 46% (6 out of 13) of the proteins tested have also agreed with the second prediction (Table 2), indicating that these proteins are dual localized. With the inclusion of the second prediction validations, we have experimentally validated the subcellular localization of 144 ngLOC predictions.

**Table 1:** **Experimental validation for ngLOC predicted proteins subcellular localization**

Protein	Prediction	Validation	Protein	Prediction	Validation
NR2F1	NUC	Yes	FKBP7	END	Yes
LMO2	NUC	Yes	ZFAN2B	END	Yes
LEF1	NUC	Yes	USMG5	MIT	Yes
U2AF1L4	NUC	No	UQCR10	MIT	Yes
KLF7	NUC	Yes	COX6B1	MIT	Yes
PHF5A	NUC	No	BRP44L	MIT	Yes
LMO1	NUC	Yes	UCP3	MIT	Yes
HMNG4	NUC	Yes	SFXN1	MIT	Yes
SCNM1	NUC	Yes	NDUFS8	MIT	Yes
SNRNP27	NUC	Yes	ATP5S	MIT	Yes
SSX3	NUC	Yes	COX7C	MIT	Yes
LMO1	NUC	Yes	MRPL30	MIT	Yes
PRKRIP1	NUC	Yes	MRPL15	MIT	Yes
HNRNPCL1	NUC	Yes	PHB	MIT	Yes
MAB21L1	NUC	Yes	MRPL53	MIT	No
VGLL2	NUC	Yes	MRPS24	MIT	Yes
AES	NUC	Yes	MRPL10	MIT	Yes
ST13	CYT	Yes	MRPL2	MIT	Yes
MLST8	CYT	Yes	COX7B	MIT	Yes
GNPDA2	CYT	Yes	MRPL51	MIT	No
CARD17	CYT	No	MRP63	MIT	Yes
RAC1	CYT	No	COQ9	MIT	Yes
FKBP1B	CYT	Yes	LGMN	LYS	Yes
SPRR2F	CYT	Yes	CTSL2	LYS	Yes
SPRR2G	CYT	Yes	MMD	LYS	Yes
NUD10	CYT	Yes	RAB7A	LYS	Yes
PCTP	CYT	No	ITM2C	LYS	Yes
PEBP1	CYT	No	DECR2	POX	Yes
RPL36AL	CYT	No	ZADH2	POX	Yes
GST5A	CYT	No	PXMP4	POX	Yes
OTUB1	CYT	Yes	TUBAL3	CSK	Yes
PCMT1	CYT	No	TMSB15A	CSK	No
PGPEP1	CYT	No	DYNLL2	CSK	No
PGEP1-2	CYT	No	ACTBL2	CSK	Yes
PMP2	CYT	No	CAPZB	CSK	Yes
PPIAL4A	CYT	No	TMSB4Y	CSK	No
UBE2K	CYT	Yes	CAPZA1	CSK	Yes
UXS1	GOL	Yes	ANKRA2	CSK	Yes
UGCG	GOL	Yes	PDLIM1	CSK	Yes
GKAP1	GOL	Yes	TRIM54	CSK	Yes
HS2ST1	GOL	Yes	PNP	CSK	Yes
GKAP1-2	GOL	Yes	CDC42EP5	CSK	Yes
GCNT2	GOL	Yes	SEP3	CSK	Yes
GABRAPL2	GOL	No	ACTRT3	CSK	Yes
ZADHHC3	GOL	Yes	NABP	PLA	Yes
ST6SIA1	GOL	Yes	GNAS	PLA	Yes
SACM1L	END	Yes	KCNIP2	PLA	Yes
SEC11A	END	Yes	MOG	PLA	Yes
SEC11C	END	Yes	CD8B	PLA	Yes
CNPY3	END	Yes	CACNG4	PLA	Yes
CNPY3-2	END	Yes	IFITM2	PLA	No
SEC61G	END	Yes	STOML3	PLA	Yes
DGAT2	END	Yes	RTP1	PLA	Yes
ASPH	END	Yes	ABHD6	PLA	Yes
MEST	END	Yes	RASD2	PLA	Yes
DOLPP1	END	Yes	TMEM68	PLA	Yes
POFUT1	END	Yes	RHOV	PLA	Yes

**Table 2:** **Experimental validation of ngLOC top second predicted proteins subcellular localization**

Protein	First Prediction	Second Prediction	Validation for First prediction	Validation for Second prediction
U2AF1L4	NUC	CYT	*	*
CARD17	CYT	NUC	*	*
RAC1	CYT	PLA	No	No
PCTP	CYT	NUC	*	*
PEBP1	CYT	PLA	No	No
RPL36AL	CYT	NUC	*	*
GST5A	CYT	NUC	*	*
PCMT	CYT	NUC	*	*
PGPEP1	CYT	NUC	*	*
PMP2	CYT	NUC	*	*
PPIAL4A	CYT	MIT	No	Yes
GABRAPL2	GOL	CSK	No	Yes
MRPL51	MIT	CYT	No	No
MRPL53	MIT	CYT	No	No
DYNLL2	CSK	NUC	*	*
TMSB4Y	CSK	CYT	*	*
TMSB15A	CSK	NUC	*	*
PXMP4	POX	PLA	Yes	No
MMD	LYS	PLA	Yes	No
RAB7A	LYS	PLA	Yes	No
SEC61G	END	MIT	Yes	Yes
DGAT2	END	PLA	Yes	No
DOLPP1	END	PLA	Yes	Yes
SEC11A	END	PLA	Yes	No
CNPY3	END	PLA	Yes	No
LMO1	NUC	MIT	Yes	Yes
LMO2	NUC	PLA	Yes	Yes
NUDT10	CYT	PLA	Yes	No
CDC42EP5	CSK	PLA	Yes	Yes
ZDHHC3	GOL	PLA	Yes	Yes

The asterisk (*) represents homogenous distribution where localization of protein was seen in both cytoplasm and nucleus.

We also looked into the correlation between the confidence score (CS) and prediction accuracy for ngLOC predictions. CS is expressed as percentage and the value can range from zero to 100. ngLOC method uses a minimum CS of 20 to make predictions (7), however we chose only a small subset of predicted proteins for validation. The CS for validated proteins ranges from 20 to 73 in this study. We divided the total number of validated proteins into two groups, low CS group (CS <46) and high CS group (CS >46); where, CS of 46 is the midpoint of the CS range for the proteins validated. Our validation results showed that 88% (50 out of 57) of the low CS group proteins were predicted accurately, compared to that of the high CS group proteins, which was 77% (44 out of 57). While these results are counter-intuitive, the high CS group contains a number of proteins that are predicted to be localized to cytoplasm, which has the highest false positive rate. Without counting the cytoplasmic proteins, the accuracies would be 92% for low CS group and 89% for the high CS group. These results demonstrate that there is no significant correlation between the CS and prediction accuracy. We presume that the lack of correlation is due to the unbalanced selection of validated proteins from a narrow range of confidence scores (see Additional file 3: Table S1), which in turn is due to feasibility (project costs limiting the sample size) and technical (PCR amplification of longer genes) issues that limited our ability to select proteins from a wider CS range for validation.Despite the lower CS range predictions for proteins localized to ER (33-65%), lysosomal (38-50%) and peroxisomal (23-39%), the validation accuracy is 100% at these locations. Similarly, plasma membrane, cytoskeletal, mitochondrial, Golgi and nuclear proteins recorded about 85% accuracy (Figure 3). Conversely, cytoplasmic proteins scored the lowest with only 45% prediction accuracy. The high false positives in this location can be attributed to the fact that cytoplasm location, being the default location for protein synthesis, lacks specific targeting signals that makes it difficult to predict. Another reason could be the dual- or multi-localization of about one-third of cytoplasmic proteins to other locations (7); where, the machine learning methods face difficulty in discriminating the cytoplasmic proteins compared to those from other locations.Overall, the experimental validations in this study prove that the ngLOC method can predict the subcellular localization of proteins at an accuracy of 82.5%, contrary to the reported accuracy of 89% (7). However, with the exclusion of the low performing cytoplasmic location (45%), the average accuracy rate jumped to 90.4% (85 out of 94). As shown in Figure 3, the accuracy is especially notable for the locations with smaller proteomes (ER, Golgi, Lysosome and Peroxisome), which are typically difficult to predict by machine learning methods. These results demonstrate the robustness, accuracy, and application in annotating the unknown subcellular localization of proteomes of eukaryotic species using the ngLOC method.

### Conclusion

This study experimentally validates and reports the accuracy of a computational method called ngLOC that predicts the subcellular localization of protein sequences in eukaryotic cells. We validated 114 human proteins that were predicted to be localized to nine distinct subcellular locations in eukaryotic cells. The overall validation accuracy rate of ngLOC method is at 82.5%, while the rate improved to 90.4% just by excluding the cytoplasmic location, compared to the overall prediction accuracy of 89%. Thus, this validation study demonstrates that ngLOC can be reliably used (with the exception of cytoplasmic location) to annotate the subcellular localization of proteins and affirms the utility of this method in large-scale annotation of newly sequenced proteomes.

## Electronic supplementary material

Additional file 1: **Primer list with the restriction sites used for gene cloning.** (DOCX 32 KB)

Additional file 2: **Predictions by ngLOC method for proteins without a known subcellular localization.** (XLSX 88 KB)

Additional file 3: Table S1: Statistics showing the spread and range of confidence scores (CS) in the predicted and validated proteins in each subcellular location. (DOCX 60 KB)

## Abbreviations

GFP: Green fluorescent protein
RFP: Red fluorescent protein
PLA: Plasma membrane
CSK: Cytoskeleton
CYT: Cytoplasm
END: Endoplasmic reticulum
GOL: Golgi complex
MIT: Mitochondria
LYS: Lysosome
POX: Peroxisome
NUC: Nucleus
UNMC: University of Nebraska Medical Center.

## References

[1] Donnes P, Hoglund A (2004). Predicting protein subcellular localization: past, present, and future. Genomics Proteomics Bioinformatics.

[2] Sprenger J, Fink JL, Teasdale RD (2006). Evaluation and comparison of mammalian subcellular localization prediction methods. BMC Bioinformatics.

[3] Emanuelsson O, Nielsen H, Brunak S, von Heijne G (2000). Predicting subcellular localization of proteins based on their N-terminal amino acid sequence. J Mol Biol.

[4] Guda C, Fahy E, Subramaniam S (2004). MITOPRED: a genome-scale method for prediction of nucleus-encoded mitochondrial proteins. Bioinformatics.

[5] Nakai K, Kanehisa M (1992). A knowledge base for predicting protein localization sites in eukaryotic cells. Genomics.

[6] Park KJ, Kanehisa M (2003). Prediction of protein subcellular locations by support vector machines using compositions of amino acids and amino acid pairs. Bioinformatics.

[7] King BR, Guda C (2007). ngLOC: an n-gram-based Bayesian method for estimating the subcellular proteomes of eukaryotes. Genome Biol.

[8] King BR, Vural S, Pandey S, Barteau A, Guda C (2012). ngLOC: software and web server for predicting protein subcellular localization in prokaryotes and eukaryotes. BMC Research Notes.

[9] Band V, Zajchowski D, Kulesa V, Sager R (1990). Human papilloma virus DNAs immortalize normal human mammary epithelial cells and reduce their growth factor requirements. Proc Natl Acad Sci U S A.

