# Supplementary material for: Experimental validation of predicted subcellular localizations of human proteins
Source: BMC Res Notes. 2014 Dec 15;7:912. doi: 10.1186/1756-0500-7-912 (PMC4301851; doi:10.1186/1756-0500-7-912)
Supplement: Supplementary file 1 — Additional file 1: Primer list with the restriction sites used for gene cloning. (DOCX 32 KB) [file 13104_2014_3411_MOESM1_ESM.docx]

**Primers list with restriction sites and their TM’s used for the cloned genes:**

LMO1-NheI-For 5'- TAT A**GC TAG C**AT GAT GGT GCT GGA CAA GGA GGA CG -3' (65.5)

LMO1-XhoI-Rev 5'- TAT A**CT CGA G**CT GAA CTT GGG ATT CAA AGG TGC CA -3' (64.4)

PHF5A-NheI-For 5'- TAT A**GCT AGC** ATG GCT AAA CAT CAT CCT GAT TTG A -3' (60.3)

PHF5A-XhoI-Rev 5'- TAT A**CTC GAG** CCT CTT CTT GAA GCC GTA TTT TTT G -3' (60.9)

KLF7-NheI-For 5'- GCC **GCT AGC** ATG GAC GTG TTG GCT AGT TAT AGT ATA -3' (63.9)

KLF7-XhoI-Rev 5'- TAT A**CT CGA G**GA TAT GTC TCT TCA TGT GGA GGG CAA G -3' (63.2)

SNRNP27-NheI-For 5'- TAT A**GC TAG C**AT GGG TCG CAG TCG CAG CCG -3' (67.5)

SNRNP27-XhoI-Rev 5'- GCC **CTC GAG** TGC AAT GAA ATC CAA AGG TCT GTT G -3' (64.9)

HMNG4-NheI-For 5'- TAT A**GC TAG C**AT GCC CAA GAG AAA GGC AAA AGG AGA T -3' (64.1)

HMNG4-XhoI-Rev 5'- TAT A**CT CGA G**CT TGG CAT CCC CAG TGC CTT C -3' (65.6)

SSX3-NheI-For 5'- TAT A**GC TAG C**AT GAA CGG AGA TGA CAC CTT TGC AAG G -3' (64.2)

SSX3-XhoI-Rev 5'- TAT A**CT CGA G**CT CAT CAT CTT CCT CAG GAT CGC TGA T -3' (64)

HNRNPCL1-NheI-For 5'- TAT A**GC TAG C**AT GGC CAG CAA CGT TAC CAA CAA GA -3' (64.6)

HNRNPCL1-HindIII-Rev 5'- TAT A**AA GCT T**AG AGT CAT CCT GGC CAT TGG TGC TG -3' (63.4)

PRKRIP1-BglII-For 5'- TAT A**AG ATC T**AT GGC TAG CCC AGC CGC CTC CTC GG -3' (67.6)

PRKRIP1-BamHI-Rev 5'- TAT A**GG ATC C**TC GCC CCA TGG TGA AAC TGG GCA C -3' (67.5)

PPM1M-NheI-For 5'- TAT A**GC TAG C**AT GCA CCT CAA TGG CCG CTG CAT CT -3' (67.3)

PPM1M -XhoI-Rev 5'- TAT A**CT CGA G**GT GGT CAC TGC TCT CTT GGC CCT GA -3' (67.3)

ST8SIA1-NheI-For 5'- TAT A**GC TAG C**AT GAG CCC CTG CGG GCG GGC -3' (70.9)

ST8SIA1-XhoI-Rev 5'- GCC **CTC GAG** GGA AGT GGG CTG GAG TGA GGT ATC TTC -3' (69.1)

PXMP4-NheI-For 5'- TAT **AGC TAG C**AT GGC AGC CCC GCC GCA GCT AA -3' (69.7)

PXMP4-XhoI-Rev 5'- GCC **CTC GAG** ATT GGA GGG ACG GCT CTT GTT ATA G -3' (66)

MMD-NheI-For 5'- TAT A**GC TAG C**AT GCG GTT CAA GAA TCG ATT CCA G -3' (62.2)

MMD-XhoI-Rev 5'- TAT A**CT CGA G**TA AAT GCC GCA TAA AGT CCG TAG GA -3' (62.3)

RAB7A-NheI-For 5'- TAT A**GC TAG C**AT GAC CTC TAG GAA GAA AGT GTT GCT GAA -3' (63.1)

RAB7A-XhoI-Rev 5'- TAT A**CT CGA G**GC AAC TGC AGC TTT CTG CCG AG -3' (65.4)

ITM2C-NheI-For 5'- TAT A**GC TAG C**AT GGT GAA GAT TAG CTT CCA GCC CG -3' (64.3)

ITM2C-XhoI-Rev 5'- TAT A**CT CGA G**CA CCA CCC CGC AGA TGA GCG TCT -3' (68.3)

SPRR2F-NheI-For 5'- TAT A**GC TAG C**AT GTC TTA TCA ACA GCA GCA GTG CA -3' (63.1)

SPRR2F-XhoI-Rev 5'- TAT A**CT CGA G**CT TGC TCT TGG GTG GAC ACT TTG G -3' (64.8)

SPRR2G-NheI-For 5'- TAT A**GC TAG C**AT GTC TTA CCA GCA GCA GCA GTG -3' (63.6)

SPRR2G-XhoI-Rev 5'- TAT A**CT CGA G**CT TGC TCT TGG GTG GAT ACT TCT G -3' (62.8)

RPL36AL -NheI-For 5'- TAT A**GC TAG C**AT GGT TAA CGT CCC TAA AAC CCG -3' (62)

RPL36AL -XhoI-Rev 5'- TAT A**CT CGA G**GA ACT GGA TCA CTT GGC CCT TTC T -3' (63.9)

PCTP -NheI-For 5'- TAT A**GC TAG C**AT GGA GCT GGC CGC CGG AAG -3' (67.5)

PCTP -XhoI-Rev 5'- GCC **CTC GAG** GGT TTT CTT GAG GTA GTT CTG ACA GG -3' (65.9)

GSTA5-NheI-For 5'- TAT A**GC TAG C**AT GGC AGA GAA GCC CAA GCT CCA -3' (66)

GSTA5-HindIII-Rev 5'- GCC **AAG CTT** AAA CCT GAA AAT CTT CCT TGC TTC TTC T -3' (62.9)

PEBP1 -NheI-For 5'- TAT A**GC TAG C**AT GCC GGT GGA CCT CAG CAA GT -3' (66.3)

PEBP1 -XhoI-Rev 5'- TAT A**CT CGA G**CT TCC CAG ACA GCT GCT CGT ACA GT -3' (65.9)

PCMT1-NheI-For 5'- TAT A**GC TAG C**AT GGC CTG GAA ATC CGG CGG -3' (66.2)

PCMT1 -XhoI-Rev 5'- TAT A**CT CGA G**CT TCC ACC TGG ACC ACT GCT TTT C -3' (64.6)

NUD10-NheI-For 5'- TAT A**GC TAG C**AT GAA GTG CAA ACC CAA CCA GAC AC -3' (63.7)

NUD10 -XhoI-Rev 5'- TAT A**CT CGA G**GG GAT CGC TAT CTG GCG AGG ATG -3' (65.2)

OTUB1-NheI-For 5'- TAT A**GC TAG C**AT GGC GGC GGA GGA ACC TCA G -3' (66.8)

OTUB1-XhoI-Rev 5'- GCC **CTC GAG** TTT GTA GAG GAT ATC GTA GTG TCC AGG -3' (64.9)

PGPEP1-NheI-For 5'- TAT A**GC TAG C**AT GGA GCA GCC GAG GAA GGC -3' (65.7)

PGPEP1-XhoI-Rev 5'- TAT A**CT CGA G**GT GTT TGT GGC AAT AGT TGA TTT TGC CC -3' (63.5)

PMP2-NheI-For 5'- TAT A**GC TAG C**AT GAG CAA CAA ATT CCT GGG CAC CT -3' (64.7)

PMP2-XhoI-Rev 5'- TAT A**CT CGA G**GA CCT TCT CAT AGA TTC TGG TGC ACA C -3' (63.2)

UBE2K-NheI-For 5'- TAT A**GC TAG C**AT GGC CAA CAT CGC GGT GCA -3' (66.2)

UBE2K-XhoI-Rev 5'- GCC **CTC GAG** GTT ACT CAG AAG CAA TTC TGT TGC AG -3' (65.3)

PPIAL4A-NheI-For 5'- TAT A**GC TAG C**AT GGT CAA CTC CGT CGT CTT TTT TGA C -3' (63)

PPIAL4A-XhoI-Rev 5'- TAT A**CT CGA G**GA ATT GTC CAC AGT CAG CAA TGG TGA T -3' (63.7)

KCNIP2-NheI-For 5'- TAT A**GC TAG C**AT GCG GGG CCA GGG CCG CAA -3' (70.9)

KCNIP2-XhoI-Rev 5'- GCC **CTC GAG** GAT GAC ATT GTC AAA GAG CTG CAT GGA -3' (63.7)

MOG-NheI-For 5'- TAT A**GC TAG C**AT GGC AAG CTT ATC AAG ACC CTC TCT GC -3' (64.9)

MOG-XhoI-Rev 5'- TAT A**CT CGA G**GA AGG GAT TTC GTA GCT CTT CAA GGA A -3' (63)

CD8B-NheI-For 5'- TAT A**GC TAG C**AT GCG GCC GCG GCT GTG G -3' (69)

CD8B-XhoI-Rev 5'- GCC **CTC GAG** TTT GTA AAA TTG TTT CAT GAA ACG AAG CCG -3' (64.4)

CACNG4-NheI-For 5'- TAT A**GC TAG C**AT GGT GCG ATG CGA CCG CGG -3' (67.8)

CACNG4-HindIII-Rev 5'- TAT A**AA GCT T**CA CAG GGG TCG TCC GTC GGT T -3' (65.1)

IFITM2-NheI-For 5'- TAT A**GC TAG C**AT GAA CCA CAT TGT GCA AAC CTT CTC TCC -3' (64.1)

IFITM2-XhoI-Rev 5'- TAT A**CT CGA G**TC GCT GGG CCT GGA CGA CCA -3' (67.8)

STOML3-NheI-For 5'- TAT A**GC TAG C**AT GGA TTC TAG GGT GTC TTC ACC TGA -3' (63)

STOML3-XhoI-Rev 5'- TAT A**CT CGA G**GG CTT TAT TTG GAA GCT TCT TGT GGT -3' (62.9)

RTP1-NheI-For 5'- TAT A**GC TAG C**AT GAG GAT TTT TAG ACC GTG GAG AC -3' (61.4)

RTP1-XhoI-Rev 5'- TAT A**CT CGA G**TA CGG AGC TAC GGA AAG AGA ACT GC -3' (63.4)

ABHD6-NheI-For 5'- TAT A**GC TAG C**AT GGA TCT TGA TGT GGT TAA CAT GTT TG -3' (60.8)

ABHD6-XhoI-Rev 5'- TAT A**CT CGA G**GT CCA GCT TCT TGT TGT TGT CTG T -3' (62.8)

RASD2-NheI-For 5'- TAT A**GC TAG C**AT GAT GAA GAC TTT GTC CAG CGG G -3' (63.3)

RASD2-XhoI-Rev 5'- TAT A**CT CGA G**CT GGA TGG TGC ACT TGT CCC TCT -3' (65.5)

TMEM68-NheI-For 5'- TAT A**GC TAG C**AT GAT AGA CAA AAA TCA AAC CTG TGG TG -3' (60.8)

TMEM68-XhoI-Rev 5'- GCC **CTC GAG** ATG AAA ACG TTC TAA CAA AGC ACT C -3' (63.1)

RHOV-NheI-For 5'- TAT A**GC TAG C**AT GCC GCC GCG GGA GCT GAG -3' (69.1)

RHOV-XhoI-Rev 5'- GCC **CTC GAG** AAC GAA GCA GAA GAA CTT CTT CCA GCG -3' (67.3)

SEC61G-NheI-For 5'- TAT A**GC TAG C**AT GGA TCA GGT AAT GCA GTT TGT TG -3' (61)

SEC61G-XhoI-Rev 5'- GCC **CTC GAG** GCC ACC AAC AAT GAT GTT ATT AAT A -3' (62.7)

DGAT2-NheI-For 5'- TAT A**GC TAG C**AT GAA GAC CCT CAT AGC CGC CTA CT -3' (64.6)

DGAT2-XhoI-Rev 5'- TAT A**CT CGA G**GT TCA CCT CCA GGA CCT CAG TCT C -3' (64.6)

ASPH-NheI-For 5'- TAT A**GC TAG C**AT GGC TGA AGA TAA AGA GAC AAA GC -3' (60.6)

ASPH-XhoI-Rev 5'- TAT A**CT CGA G**AG TAT CTG GTG GTA CTT CCT GCT GT -3' (62.8)

MEST-NheI-For 5'- TAT A**GC TAG C**AT GGT GCG CCG AGA TCG CCT -3' (67)

MEST-HindIII-Rev 5'- GCC **AAG CTT** GAA GGA GTT GAT GAA GCC CAT ATA TGC -3' (64.3)

DOLPP1-NheI-For 5'- TAT A**GC TAG C**AT GGC AGC GGA CGG ACA GTG C -3' (67.2)

DOLPP1-XhoI-Rev 5'- TAT A**CT CGA G**CT GCA GTT TCG TCC CCA GCT TG -3' (65.3)

POFUT1-NheI-For 5'- TAT A**GC TAG C**AT GGG CGC CGC CGC GTG G -3' (70.7)

POFUT1-XhoI-Rev 5'- TAT A**CT CGA G**GA ACT CGT CCC GCA GCT TAG GGG G -3' (67.8)

FKBP7-NheI-For 5'- TAT A**GC TAG C**AT GCC AAA AAC CAT GCA TTT CTT ATT C -3' (60.5)

FKBP7-XhoI-Rev 5'- GCC **CTC GAG** TAG TTC ATC GTG TTG GTA TAC ATT GT -3' (63.1)

ZFAN2B-NheI-For 5'- TAT A**GC TAG C**AT GGA GTT TCC GGA CCT CGG CG -3' (66.3)

ZFAN2B-XhoI-Rev 5'- TAT A**CT CGA G**GC ACA GGC TGC AGT TGG ACG G -3' (67)

COX6B1-NheI-For 5'- TAT A**GC TAG C**AT GGC GGA AGA CAT GGA GAC CAA AA -3' (64.3)

COX6B1-XhoI-Rev 5'- TAT A**CT CGA G**GA TCT TCC CGG GAA ACG TGC CTT -3'

BRP44L-NheI-For 5'- TAT A**GC TAG C**AT GGC GGG CGC GTT GGT G -3' (67.4)

BRP44L-XhoI-Rev 5'- GCC **CTC GAG** TGC AGA TGC CGT TTT AGT CAT CTC GTG -3' (67.3)

UCP3-NheI-For 5'- TAT A**GC TAG C**AT GGT TGG ACT GAA GCC TTC AGA CG -3' (64.3)

UCP3-XhoI-Rev 5'- TAT A**CT CGA G**AA ACG GTG ATT CCC GTA ACA TCT GGA -3' (63.3)

SFXN1-BglII-For 5'- GCC **AGA TCT** ATG TCT GGA GAA CTA CCA CCA AAC ATT -3' (63.1)

SFXN1-BamHI-Rev 5'- TAT A**GG ATC C**CA ATC CCT TAT TGA AGT ACA CGC GT -3' (62.5)

NDUFS8-NheI-For 5'- TAT A**GC TAG C**AT GCG CTG CCT GAC CAC GCC TAT -3' (67.5)

NDUFS8-XhoI-Rev 5'- GCC **CTC GAG** CCG ATA CAA GTA GTC AGC CTG GAT -3' (67.4)

ATP5S-NheI-For 5'- TAT A**GC TAG C**AT GTG CTG TGC GGT CTC TGA GCA -3' (65.9)

ATP5S-XhoI-Rev 5'- GCC **CTC GAG** CTT CAA TTG TAA TTT TAG TTC CAG AG -3' (61.6)

COX7C-NheI-For 5'- TAT A**GC TAG C**AT GTT GGG CCA GAG CAT CCG GA -3' (66.3)

COX7C-XhoI-Rev 5'- GCC **CTC GAG** TGT TTT AAG CAG TTG GTG TCT TAC TAC -3' (63.6)

MRPL30-NheI-For 5'- TAT A**GC TAG C**AT GGC TGG GAT TTT GCG CTT AGT AGT T -3' (64.1)

MRPL30-XhoI-Rev 5'- TAT A**CT CGA G**GG ACT CAT GTG CTT TCT GCT CCA CA -3' (65.1)

MRPL15-NheI-For 5'- TAT A**GC TAG C**AT GGC CGG TCC CTT GCA G -3' (65.1)

MRPL15-XhoI-Rev 5'- GCC **CTC GAG** TGA GGT ATA ATA CTT AAG GAG ATT TTC ATC -3' (61.2)

PHB-NheI-For 5'- GCC **GCT AGC** ATG GCT GCC AAA GTG TTT GAG TCC -3' (68.1)

PHB-XhoI-Rev 5'- TAT A**CT CGA G**CT GGG GCA GCT GGA GGA GCA -3' (68.2)

MRPL53-NheI-For 5'- TAT A**GC TAG C**AT GGC AGC TGC CTT GGC TCG -3' (66.3)

MRPL53-XhoI-Rev 5'- TAT A**CT CGA G**GC GAC CAG TAT CAG CGC CCG -3' (66.8)

MRPS24-NheI-For 5'- TAT A**GC TAG C**AT GGC GGC CTC CGT GTG CAG -3' (67.7)

MRPS24-XhoI-Rev 5'- GCC **CTC GAG** GAG GTA CTT ATA CAC AAC CTT TGA GGG C -3' (66.5)

MRPL10-NheI-For 5'- TAT A**GC TAG C**AT GGC TGC GGC CGT GGC G -3' (69)

MRPL10-XhoI-Rev 5'- TAT A**CT CGA G**CG AGT CCG GAA CAG TGT CAG GAT CTG G -3' (66.7)

MRPL2-NheI-For 5'- TAT A**GC TAG C**AT GGC CCT GTG CGC ACT GAC -3' (66.3)

MRPL2-XhoI-Rev 5'- TAT A**CT CGA G**GC TTT GGG CAG AAG CAG AAG GCA G -3' (66.2)

COX7B-NheI-For 5'- TAT A**GC TAG C**AT GTT TCC CTT GGT CAA AAG CGC A -3' (63.9)

COX7B-XhoI-Rev 5'- TAT A**CT CGA G**CT GAT TCC TCC ATT CCT TTG GGG TA -3' (63.5)

MRPL51-NheI-For 5'- TAT A**GC TAG C**AT GGC AGG GAA CCT CTT ATC CGG -3' (64.4)

MRPL51-XhoI-Rev 5'- TAT A**CT CGA G**TC GAA ACT TCC CAT GTC GGT TAA AG -3' (61.6)

MRP63-NheI-For 5'- TAT A**GC TAG C**AT GTT CCT GAC TGC GCT CCT CTG -3' (64.6)

MRP63-XhoI-Rev 5'- TAT A**CT CGA G**GG ACC ATT TCT TGG TGA CAT TGA GAT -3' (62.4)

COQ9-NheI-For 5'- TAT A**GC TAG C**AT GGC GGC GGC GGC GGT ATC -3' (69.2)

COQ9-XhoI-Rev 5'- TAT A**CT CGA G**CC GAC GCT GGT TTA GAC CTG TCA AGT T -3' (65.8)

MAB21L1-NheI-For 5'- TAT A**GC TAG C**AT GAT TGC GGC CCA GGC CAA GCT -3' (68.1)

MAB21L1-HindIII-Rev 5'- GCC **AAG CTT** AAG TTT TTC CAA ACT TTT CGG GTT GGT -3' (63.7)

VGLL2-NheI-For 5'- GCC **GCT AGC** ATG AGC TGT CTG GAT GTT ATG TAC CAA -3' (65.8)

VGLL2-XhoI-Rev 5'- TAT A**CT CGA G**GC TCA GGA GGG ATG CAC CAC AGA -3' (66.6)

AES-NheI-For 5'- TAT A**GC TAG C**AT GAT GTT TCC ACA AAG CAG GCA TTC G -3' (63.7)

AES-XhoI-Rev 5'- TAT A**CT CGA G**AT CCG ACT TCT CGC CAT CAT CCT C -3' (63.5)

DYNLL2-NheI-For 5'- TAT A**GC TAG C**AT GTC TGA CCG GAA GGC AGT GAT CAA GAA -3' (65.4)

DYNLL2-XhoI-Rev 5'- TAT A**CT CGA G**GC CTG ACT TGA AGA GGA GGA TTG CAA -3' (64.8)

ACTBL2-NheI-For 5'- TAT A**GC TAG C**AT GAC TGA CAA TGA GCT GTC TGC CTT -3' (63.9)

ACTBL2-XhoI-Rev 5'- TAT A**CT CGA G**GA AAC ATT TTC TGT GAA CGA TAG GAG G -3' (61.3)

CAPZB-NheI-For 5'- TAT A**GC TAG C**AT GAG TGA TCA GCA GCT GGA CTG T -3' (64)

CAPZB-XhoI-Rev 5'- TAT A**CT CGA G**GC ATT GCT GCT TTC TCT TCA AAG CC -3' (63.3)

TMSB4Y-NheI-For 5'- TAT A**GC TAG C**AT GTC TGA CAA ACC TGG TAT GGC T -3' (62.8)

TMSB4Y-XhoI-Rev 5'- TAT A**CT CGA G**AG ATT CGC CTG CTT GCC TCT CC -3' (64.6)

CAPZA1-NheI-For 5'- TAT A**GC TAG C**AT GGC CGA CTT CGA TGA TCG TGT GT -3' (65.2)

CAPZA1-XhoI-Rev 5'- TAT A**CT CGA G**AG CAT TCT GCA TTT CTT TGC CAA TCT TG -3' (62.3)

ANKRA2-NheI-For 5'- TAT A**GC TAG C**AT GGA TAC ATC AAC AAA TCT GGA TAT TG -3' (59.2)

ANKRA2-XhoI-Rev 5'- TAT A**CT CGA G**CT CCT TGA TAT TTT GAA GCA GCT TCA AC -3' (61.9)

PDLIM1-NheI-For 5'- TAT A**GC TAG C**AT GAC CAC CCA GCA GAT AGA CCT CCA G -3' (65.6)

PDLIM1-XhoI-Rev 5'- TAT A**CT CGA G**CT TGG GGA ACA CAG TGA CCA CTT CAT AA -3' (64.5)

TRIM54-NheI-For 5'- GCC **GCT AGC** ATG AAC TTC ACA GTG GGT TTC AAG CC -3' (67.3)

TRIM54-XhoI-Rev 5'- TAT A**CT CGA G**AG GCC CAT CCG GCC GCT CTT -3' (68.1)

PNP-NheI-For 5'- TAT A**GC TAG C**AT GGA GAA CGG ATA CAC CTA TGA AGA -3' (61.7)

PNP-XhoI-Rev 5'- TAT A**CT CGA G**AC TGG CTT TGT CAG GGA GTG GAA T -3' (63.8)

CDC42EP5-NheI-For 5'- TAT A**GC TAG C**AT GCC CGT GCT GAA GCA GCT GG -3' (67.2)

CDC42EP5-HindIII-Rev 5'- TAT A**AA GCT T**GA GGC CGA TGA CGT CGT TCA GCT C -3' (64.8)

SEPT3-NheI-For 5'- TAT A**GC TAG C**AT GTC CAA AGG GCT CCC AGA GAC CA -3' (66.3)

SEPT3-XhoI-Rev 5'- TAT A**CT CGA G**TT CAG CAG TGG GGC AGG GGG T -3' (67.8)

ACTRT3-NheI-For 5'- TAT A**GC TAG C**AT GAA CCA CTG CCA GCT ACC GGT G -3' (66)

ACTRT3-XhoI-Rev 5'- GCC **CTC GAG** GAA GCA TCT TTG GTG TAC TAT GTT GGG TC -3' (66.6)

ZADH2-NheI-For 5'- TAT A**GC TAG C**AT GCT GCG GCT GGT GCC CAC -3' (68.1)

ZADH2-HindIII-Rev 5'- GCC **AAG CTT** CAG CTT ACT GTT GAC AGA GTG AGG TAA TTC -3' (64.4)

GABARAPL2-NheI-For 5'- GCC **GCT AGC** ATG AAG TGG ATG TTC AAG GAG GAC CA -3' (67.5)

GABARAPL2-XhoI-Rev 5'- GCC **CTC GAG** GAA GCC AAA AGT GTT CTC TCC GCT -3' (68.2)

HS2ST1-NheI-For 5'- GCC **GCT AGC** ATG GGG CTC CTC AGG ATT ATG ATG -3' (67)

HS2ST1-XhoI-Rev 5'- GCC **CTC GAG** GTT CGA CTT AGG GTA AAT CTT TTC AT -3' (63.3)

GCNT2-NheI-For 5'- GCC **GCT AGC** ATG ATG GGC TCT TGG AAG CAC TGT C -3' (68.5)

GCNT2-XhoI-Rev 5'- GCC **CTC GAG** AAA ATA CCA GCT GGG TTG TAT CGC AG -3' (66.5)

GKAP1-NheI-For 5'- GCC **GCT AGC** ATG GCC TCA GCA GTA CTT AGT TCT GTT -3' (67.1)

GKAP1-XhoI-Rev 5'- GCC **CTC GAG** CCT ACA CTG GTC GGA TTC AGA GTT TC -3' (67.2)

SEC11A-NheI-For 5'- GCC **GCT AGC** ATG CTG TCT CTA GAC TTT TTG GAC GAT G -3' (65.9)

SEC11A-XhoI-Rev 5'- GCC **CTC GAG** CTC ACG ATG AAC CAG CAC GAA TAA AC -3' (66.6)

SEC11C-NheI-For 5'- TAT A**GC TAG C**AT GGT GCG TGC GGG CGC C -3' (69.1)

SEC11C-XhoI-Rev 5'- GCC **CTC GAG** GGA TTC ACG TTT TAG TAA CAC ATA TGC -3' (63.7)

CNPY3-NheI-For 5'- TAT A**GC TAG C**AT GGA TTC AAT GCC TGA GCC CGC GT -3' (67.1)

CNPY3-XhoI-Rev 5'- TAT A**CT CGA G**GA GCT CAT CAG GGG GGC TGT GTG TG -3' (67.8)

TMSB15A-NheI-For 5'- GCC **GCT AGC** ATG AGT GAT AAG CCA GAC TTG TCG GA -3' (67.1)

TMSB15A-XhoI-Rev 5'- GCC **CTC GAG** TGA TGT TTG AAC ACA CTC TTT CTC -3' (63)

FKBP1B-NheI-For 5'- TAT A**GC TAG C**AT GGG CGT GGA GAT CGA GAC CAT C -3' (65.4)

FKBP1B-XhoI-Rev 5'- GCC **CTC GAG** CTC TAA GTT GAG CAG CTC CAC GTC -3' (68)

MLST8-NheI-For 5'- TAT A**GC TAG C**AT GAA CAC CTC CCC AGG CAC GG -3' (66.5)

MLST8-XhoI-Rev 5'- TAT A**CT CGA G**GC CCA GCA CAC TGT CAT TGA AGG C -3' (66.2)

GNPDA2-NheI-For 5'- GCC **GCT AGC** ATG AGG CTT GTA ATT CTT GAT AAC TAT G -3' (62.4)

GNPDA2-XhoI-Rev 5'- GCC **CTC GAG** GTT TCC ATC TTT CAT ACT GAA TAG TGG -3' (63.3)

CARD17-NheI-For 5'- TAT A**GC TAG C**AT GGC CGA CAA GGT CCT GAA GGA -3' (65.6)

CARD17-XhoI-Rev 5'- GCC **CTC GAG** GGA AGG AAG TAC TAT TTG AGA ATC TTG -3' (63.3)

RAC1-NheI-For 5'- TAT A**GC TAG C**AT GCA GGC CAT CAA GTG TGT GGT -3' (65)

RAC1-XhoI-Rev 5'- TAT A**CT CGA G**CA ACA GCA GGC ATT TTC TCT TCC TC -3' (63.3)

UQCR10-NheI-For 5'- TAT A**GC TAG C**AT GGC GGC CGC GAC GTT GAC TTC -3' (68)

UQCR10-XhoI-Rev 5'- GCC **CTC GAG** CTT GTT CTC ATA CTT GTG CTT GAT G -3' (64.3)

U2AF1L4-NheI-For 5'- GCC **GCT AGC** ATG GCT GAA TAT TTA GCT TCG ATA TTC G -3' (63.7)

U2AF1L4-XhoI-Rev 5'- TAT A**CT CGA G**CA TTA TGG AGC CCG GGA GCC TGG -3' (67.3)

GNAS-NheI-For 5'- TAT A**GC TAG C**AT GGG CTG CCT CGG GAA CAG TAA G -3' (65.8)

GNAS-XhoI-Rev 5'- TAT A**CT CGA G**GA GCA GCT CGT ACT GAC GAA GGT G -3' (65)

UGCG-BglII-For 5'- GCC**AGATCT** ATG GCG CTG CTG GAC CTG G -3' (67.6)

UGCG-BamHI-Rev 5'- GCC**GGATCC** TA CAT CTA GGA TTT CCT CTG CTG -3' (63.7)

ZDHHC3-BglII-For 5'- GCC**AGATCT** ATG ATG CTT ATC CCC ACC CAC CAC TTC CG -3' (67.7 ºC)

ZDHHC3-BamHI-Rev 5'- GCC**GGATCC** GA CCA CAT ACT GGT ACG GGT CTG -3' (68.1 ºC)

CTSL2-BglII-For 5'- GCC**AGATCT** ATG AAT CTT TCG CTC GTC CTG GCT G -3' (65.2)

CTSL2-BamHI-Rev 5'- GCC**GGATCC** CA CAT TGG GGT AGC TGG CTG -3' (69.1)

LEF1-NheI-For 5'- TATA**GCTAGC** ATG CCC CAA CTC TCC GGA GG -3' (65.3)

LEF1-HindIII-Rev 5'- GCC**AAGCTT** GA TGT AGG CAG CTG TCA TTC TTG -3' (63.9)

LMO2-NheI-For 5'- GCC**GCTAGC** ATG TCC TCG GCC ATC GAA AG -3' (67.4)

LMO2-XhoI-Rev 5'- GCC**CTCGAG** TAT CAT CCC ATT GAT CTT AGT CCA C -3' (63.3)

SCNM1-NheI-For 5'- TAT A**GC TAG C**AT GTC TTT CAA GAG GGA AGG AGA CG -3' (62.5)

SCNM1-XhoI-Rev 5'- TAT A**CT CGA G**GT CCA AGG GGA GAT CAG GTG GTT -3' (65.1)
