# Supplementary material for: Experimental validation of predicted subcellular localizations of human proteins
Source: BMC Res Notes. 2014 Dec 15;7:912. doi: 10.1186/1756-0500-7-912 (PMC4301851; doi:10.1186/1756-0500-7-912)
Supplement: Supplementary file 3 — Additional file 3: Table S1: Statistics showing the spread and range of confidence scores (CS) in the predicted and validated proteins in each subcellular location. (DOCX 60 KB) [file 13104_2014_3411_MOESM3_ESM.docx]

**Table S1**. Statistics showing the spread and range of confidence scores (CS) in the predicted and validated proteins in each subcellular location

| **Subcellular Location** | **CS spread of all proteins predicted by ngLOC** | **CS spread of validated proteins** | **CS range** |
| --- | --- | --- | --- |
| CSK-Cytoskeleton | 11.3-82.8 | 22.8–73.2 | 50.4 |
| CYT-Cytoplasm | 10.0-77.4 | 35.5–72.7 | 37.2 |
| END- Endoplasmic reticulum | 10.5-74.5 | 24.7-68.3 | 43.6 |
| GOL- Golgi | 10.5-65.8 | 20.3–48.1 | 27.8 |
| LYS- Lysosome | 11.3-64.7 | 38.7-50.0 | 11.3 |
| MIT- Mitochondria | 10.5-85.7 | 33.3–65.1 | 31.8 |
| NUC- Nucleus | 10.5-80.9 | 37.6–70.8 | 33.2 |
| PLA- Plasma Membrane | 10.6-81.9 | 40.7-63.2 | 22.5 |
| POX- Peroxisomal | 12.8-68.7 | 23.6-39.9 | 16.3 |
